# Widening Genetic Diversity Using Embryo Rescue in Cucurbit Crops: A Review

**DOI:** 10.3390/plants13101320

**Published:** 2024-05-10

**Authors:** Chinreddy Subramanyam Reddy, Sahithi Ramireddy, Umesh K. Reddy

**Affiliations:** Department of Biology, Gus R. Douglass Institute, West Virginia State University, Institute, WV 25112, USA; subramanyam.chinreddy@wvstateu.edu (C.S.R.); sramireddy@wvstateu.edu (S.R.)

**Keywords:** embryo rescue, cucurbits, genetic diversity, in vitro culture

## Abstract

Embryo rescue is a vital technique in cucurbit breeding and propagation, addressing challenges such as embryo abortion, poor seed viability, and incompatibility barriers. This method involves the excision of immature embryos from seeds followed by their in vitro culture on a nutrient medium, providing an environment conducive to their growth and development. In cucurbits, embryo rescue has been extensively utilized to overcome barriers to hybridization, enabling the production of interspecific and intergeneric hybrids with desired traits. Various factors, including genotype, developmental stage of embryos, and culture conditions, influence the success of embryo rescue in cucurbits. Optimal nutrient formulations, growth regulators, and culture techniques are critical for promoting embryo germination, shoot elongation, and subsequent plantlet establishment. Additionally, embryo rescue facilitates the recovery of valuable genetic material from wild and exotic cucurbit species, expanding genetic diversity and developing novel cultivars with improved traits such as disease resistance, yield, and quality. This review highlights the principles, applications, and advancements in embryo rescue technology in cucurbits, emphasizing its significance in cucurbit breeding programs and crop improvement efforts.

## 1. Introduction

The Cucurbitaceae, commonly known as the pumpkin family, stands as a cornerstone in the realm of vegetables and fruits, boasting a rich diversity critical for human diets worldwide [1]. Comprising an impressive array of 130 genera and 965 species [2], it holds the distinction of being the most varied member of the vegetable plant family cultivated globally, thriving across a spectrum of environmental conditions [3]. These versatile plants are revered for their multifaceted contributions, offering not only essential nourishment but also serving as prized ornamental additions to landscapes [4,5]. From the humble pumpkin to the vibrant array of squashes, cucumbers, and melons, each member of the Cucurbitaceae family adds its unique flavor and nutritional profile to the global culinary repertoire. Moreover, their economic significance cannot be overstated, as they represent valuable commodities in agricultural markets, supporting livelihoods and economies around the world.

Despite their significance in agriculture, cucurbit breeding confronts several challenges, such as barriers to interspecific and intergeneric hybridization, embryo abortion, and poor seed viability [5,6,7]. These obstacles impede the utilization of desirable traits from wild and exotic cucurbit species, constraining the genetic diversity accessible for crop enhancement [8]. Embryo rescue (ER) is a crucial technique to address these challenges [9,10]. It involves recovering embryos from seeds that fail to germinate or develop, followed by in vitro culture on a nutrient medium to facilitate their growth [11,12,13]. This method not only aids in retrieving valuable genetic material but also allows the production of hybrids with unattainable desired traits through traditional breeding [12,14,15,16,17].

Several studies have demonstrated the efficacy of embryo rescue in cucurbits, showcasing its potential in addressing breeding challenges and expanding the genetic diversity available for crop improvement [18,19,20]. For instance, Kaur et al. successfully rescued embryos from crosses between different Cucumis species, producing fertile hull-less seed traits [21]. Similarly, Rakha et al. utilized embryo rescue to overcome hybridization barriers between three species of Cucurbita (*C. moschata*, *C. ficifolia,* and *C. martinezii*) and developed novel cucurbit cultivars with improved fruit quality traits [22]. Several other researchers employed this technique for haploid plant production, Lotfi et al. [23] restoring the viability and vigor of aged or deteriorated seeds [24] and disease resistance from an interspecific *C. pepo × C. moschata* cross [19].

In addition to facilitating interspecific hybridization, embryo rescue is a valuable tool for preserving and utilizing genetic resources from wild and exotic cucurbit species [10,25]. By rescuing embryos from seeds collected from diverse germplasm sources, breeders can access novel alleles and traits that may confer valuable agronomic characteristics such as resistance to biotic and abiotic stresses, improved yield, and superior fruit quality [26,27,28].

Despite its potential, the success of embryo rescue in cucurbits depends on various factors, including the genotype of the parental plants, the developmental stage of the embryos, and the optimization of culture conditions [23,28,29]. Furthermore, advancements in tissue culture techniques, nutrient formulations, and growth regulator applications continue to enhance embryo rescue efficiency and success rate in cucurbits [30,31].

**Figure 1:**
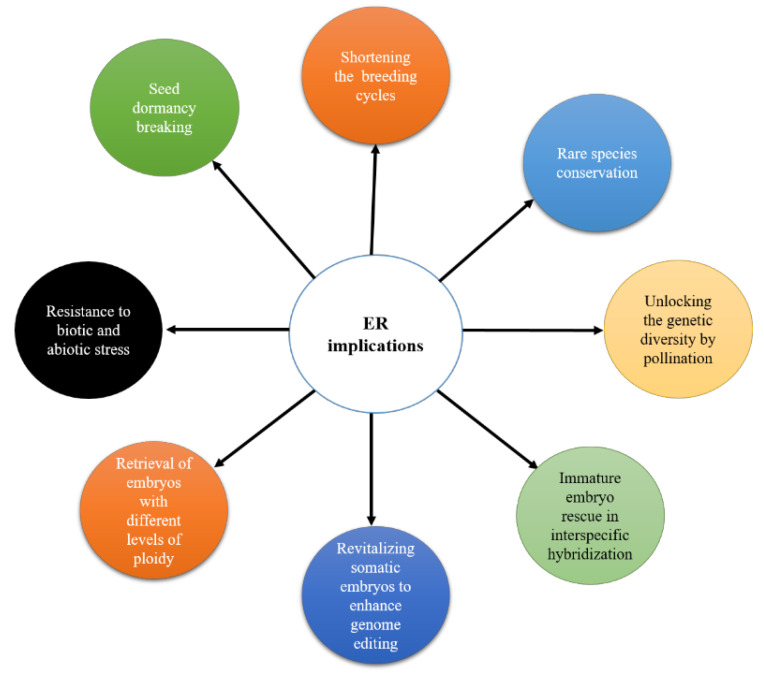
Embryo rescue and its major applications in plant breeding.

This review aims to provide an overview of cucurbits’ principles, applications (Figure 1), and advancements in embryo rescue technology, highlighting its significance in overcoming breeding barriers, expanding genetic diversity, and accelerating crop improvement efforts. Through a comprehensive investigation of the existing literature, this review aims to elucidate the potential of embryo rescue as a powerful tool in cucurbit breeding and genetic resource utilization.

## 2. Factors Leading to Immature Embryos in Plant Breeding

**Figure 2:**
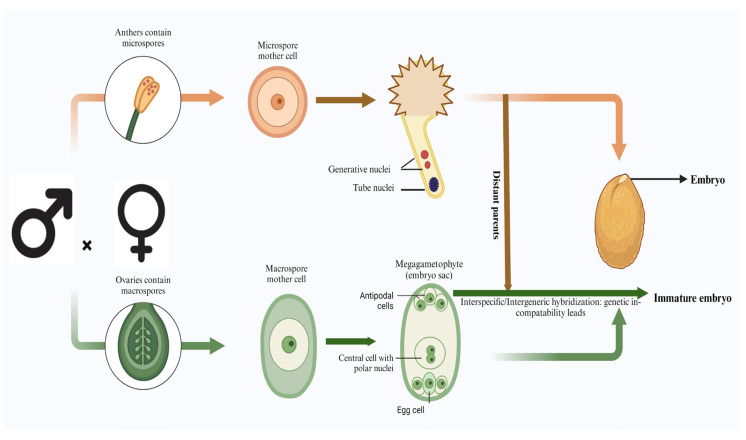
Schematic representation of general embryo formation and immature embryo formation during intraspecific, interspecific, and intergeneric hybridization.

In angiosperms, the formation of seeds undergoes a unique process termed double fertilization. Here, the male gametophyte’s sperm cell participates in a dual role: one sperm combines with an egg cell to form a diploid zygote, while simultaneously another sperm fuses with a central diploid cell, resulting from the fusion of two female polar nuclei, leading to the formation of a triploid endosperm. This collaborative effort yields both the embryo, possessing a diploid genetic makeup, and the endosperm, with a triploid constitution [32]. Encased within the protective seed coat derived from maternal tissues enveloping the egg, these nascent structures undergo continuous development, laying the foundation for future germination and growth (Figure 2).

Intraspecific, interspecific, and intergeneric hybridizations are essential for plant breeding, allowing gene transfer from wild species to cultivated crops. Crosses between different species often result in embryo-lethal mutants due to failure in endosperm formation [17]. The endosperm in flowering plants plays a vital role in facilitating nutrient and hormone transfer from maternal tissues to the embryo. Various mechanisms, such as the endosperm stable maternal and paternal count (2:1) [33], polar nuclei activation index [34], and genomic imprinting [35], contribute to hybridization barriers associated with abnormal endosperm differentiation.

The most successful technique for generating haploids in cucurbits involves using irradiated pollen [36]. However, irradiation could potentially affect the male gametophyte. One potential outcome is the alteration of genetic material within the pollen grains due to irradiation. This alteration may lead to induced seed formation without fertilization, but it could result in improper fertilization of the egg cell or the formation of a zygote with chromosomal abnormalities. Because of pseudo-fertilization, there would be no endosperm to support the growing embryo, which leads to immature embryos. Henceforth, it is essential to rescue the immature embryos from post-zygotic barriers.

## 3. Historical Background of Embryo Rescue

In the 18th century, Charles Bonnet (1720–1793) made groundbreaking strides in embryo rescue (ER) by delicately excising mature embryos from common beans and buckwheat and then adeptly transferring them into soil for growth [37]. Subsequently, researchers embarked on exploring ER’s potential, experimenting with various combinations of nutrient media—particularly those containing salt and sugar—during the pivotal period spanning from 1890 to 1904, all conducted under meticulous in vitro sterile conditions. Notably, in 1904, E. Hanning effectively utilized mature embryos from Raphanus species, nurturing them in saline media enriched with sugar to successfully cultivate seedlings. Hanning’s work underscored the critical role of high osmotic concentration, notably sucrose, and adequate nitrogen sources in fostering the robust development of seedlings from embryos [38,39]. Brown cultured isolated (*Hordeum vulgare*) embryos in a sucrose-containing mineral saline medium and revealed various organic nitrogen compounds are crucial for optimum growth [40]. His experiments also disclosed amino acids like Aspartic acid (D), Glutamic acid (E), and Asparagine (N) served as superior nitrogen sources, leading to heightened dry weight and nitrogen content in the cultured embryos. Dubard et al. explained the prominence of reserve tissues, such as endosperm and cotyledons, in embryo development, although their absolute role was elusive [41]. Furthermore, lima bean embryos without cotyledons were cultured in sucrose-containing media and showed significantly ameliorated growth [42]. Andronescu experiments with *Zea mays* described the eminence of scutellum [43]. The pioneer experiments in embryo rescue were conducted by Kurt Dieterich. By using various family plant embryos, he concluded the essential nutrients prerequisites such as C and N sources for mature and immature embryos [44]. However, the first embryo rescue experiment was conducted in 1925 by Friedrich Laibach using immature zygotic embryos from an interspecific cross between the *Lilium perenne* × *L. austriacum* [45].

## 4. Effect of Media Composition on Embryo Rescue

### 4.1. Interspecific/Intergeneric Hybridization

Interspecific hybridization within Cucurbita species presents a promising avenue for enriching genetic diversity and introducing beneficial traits from wild relatives into cultivated varieties. However, this process often faces challenges such as unsuccessful double fertilization or premature embryo abortion, hindering the successful development of hybrids. To overcome these obstacles, the regeneration of immature embryos through embryo rescue techniques is essential. Success in such interventions largely hinges on the media composition and hormones used, suggesting that with the right approach and optimized growth media, barriers to interspecific crossing can be overcome. One pioneering embryo rescue experiment conducted by Metwally et al. involved wild *Cucurbita martinezii Bailey* and domesticated *Cucurbita pepo* L., utilizing MS media supplemented with 0.01 mg/L IAA and 0.1 mg/L KIN phytohormones. This approach yielded viable plants with morphological traits intermediate between the parents, exhibiting resistance to powdery mildew and cucumber mosaic virus [18]. Another study focusing on Chinese cucumber and the Japanese parental line *Cucumis hystrix* achieved a 37% regeneration rate using minimal hormonal intervention [46]. To elucidate the genetic makeup of various cultivated and non-cultivated cucurbit species, particularly with interspecific combinations, raised embryos were rescued using MS-enriched medium supplemented with 1 mg/L nicotinic acid, 2 mg/L thiamine, 1 mg/L pyridoxine,100 mg/L myo-inositol, 15 g/L sucrose, 7 g/L agar [47].

Furthermore, research on interspecific hybridization between *Cucumis anguria* L. and *C. zeyheri Sond*. demonstrated successful embryo rescue using various MS media variants supplemented with specific nutrients and growth regulators. Ascorbic acid and coconut water were particularly beneficial for embryo growth and germination initiation, emphasizing the efficacy of targeted nutritional supplementation [48]. In another study, Rakha et al. successfully rescued immature embryos from crosses involving three cucurbit species—*Cucurbita ficifolia*, *C. moschata*, and *C. martinezii*—using MS medium supplemented with 0.1 mg/L of kinetin and 0.01 mg/L IAA [22]. Similarly, Fu et al. achieved embryo rescue in interspecific hybrids derived from *C. pepo* and *C. moschata* using MS medium supplemented with the combination of hormones mentioned in the Table 1, overcoming challenges related to parthenocarpic fruit development [25].

**Figure 3:**
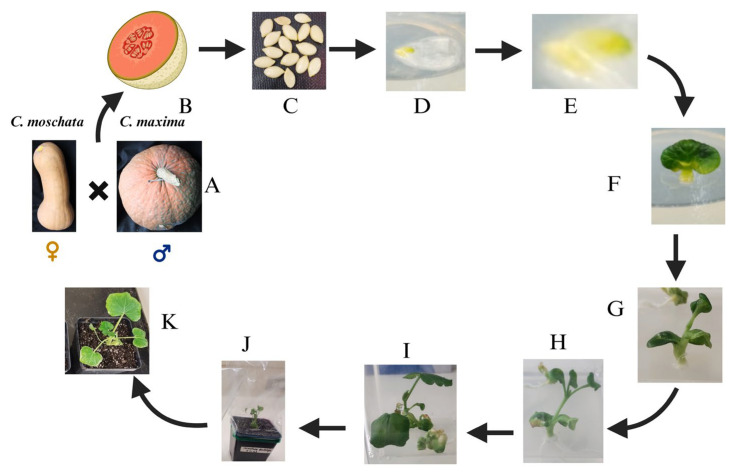
Interspecific hybridization between *Cucurbita moschata* and *Cucurbita maxima* and various stages of its immature embryo rescue. A, B & C: Seed collection and sterilization; D, E: embryo culture in MS media; F: shoot and root formation; G: cotyledonary stage; H & I: elongation; J: hardening stage; K: transferred into soil pot.

In our research, endeavors focused on enhancing carotenoid and sugar content in *Cucurbita maxima* by tapping into the genetic potential of *Cucurbita moschata butternut*. Embryo rescue from cucurbits was conducted using MS media supplemented with Zeatin and IAA in this process (Figure 3). Across these studies, the adaptability of MS medium as a base for culturing interspecific hybrids is evident. Tailoring supplementation to the specific requirements of each hybridization experiment—whether through the addition of growth regulators, vitamins, or antibiotics—has yielded successful outcomes ranging from viable plant production to enhanced disease resistance and flavor profiles. These findings underscore the potential of embryo rescue techniques in expanding genetic diversity and addressing breeding challenges in cucurbits.

### 4.2. Haploidization

In cucurbit breeding, obtaining homozygous pure lines through traditional methods is a time-consuming process, often taking 10–12 years, and achieving complete homozygosity is challenged by open pollination practices. To expedite and improve the efficiency of producing homozygous lines, in vitro techniques, particularly haploidy methodologies, offer significant advantages. These methods enable the production of plants with a haploid chromosome count, with subsequent chromosome doubling via colchicine treatment, potentially reducing the homozygotization period to 1–2 years. Among various techniques within the Cucurbitaceae family, the irradiated pollen technique emerges as the most effective for haploid induction. This approach involves generating gynogenic embryos through the use of gamma-ray irradiated pollen, followed by the crucial step of haploid embryo rescue via in vitro culture. The technique was first attempted in 1987, resulting in the successful production of haploid plants in melon and cucumber by using heart-shaped or globular-shaped embryos with E20 media along with IAA 0.01 mg/L,—saccharose 20 g/L, and agar 10 g/L [50]. Continuing the investigation with the same media composition, Sauton successfully produced gynogenetic haploids in muskmelon (*Cucumis melo* L.), studying the influence of both seasonal variations and genotype [51]. Similarly, in 1989, he applied the same technique and media composition to cucumber *(Cucumis sativus),* further expanding the scope of the study. Irradiation doses ranging from 300 to 900 gray were utilized [52,53]. Using same technique, a new method for dihaploidization in muskmelon (*Cucumis melo* L.) derived from haploids has been developed through the utilization of colchicine treatment. [54]. Across these studies, E20A medium was employed. Lofti et al. applied gamma radiation (100–400 Gy) to three cucumber cultivars and discovered that 100 Gy treatments on E20A medium resulted in viable haploid embryos, highlighting the potential of heart-shaped embryos for successful plant development [55]. Further advancements were made by Faris et al., who identified 0.1 kGy of low-dose γ-ray irradiation as optimal for haploid embryo induction in cucurbits, leading to a 7.7% plant regeneration rate from irradiated pollen-induced embryos using E20A media and developed plants grown on MS media [56]. Lofti et al. also pioneered protocols for generating haploid and doubled haploid (DH) melon plants from virus-resistant hybrids, utilizing post-pollination with irradiated pollen and a 10-day seed culture in a liquid medium before embryo excision for cultivation and further culture in E20A [57]. Sztangret et al. contributed significantly by successfully generating haploid plants and embryos from hybrid cucumber varieties, with approximately 45–48% of embryos differentiating into stable haploid plants [58]. Claveria et al. advanced the technique further by generating homozygous doubled haploid lines (DHLs) through in vitro rescue of parthenogenic embryos induced by pollen irradiated at 500 Gy, achieving an 83% success rate in converting embryos to plants. The study utilized a modified E20A medium, termed E20 H8, supplemented with specific components: 7.9 mM CaCl_2_·2H_2_O, 0.17 mM CoCl_2_·6H_2_O, 0.10 mM FeEDTA, 20 g/L sucrose, and 8 g/L Bac-to-agar. The pH was adjusted to 5.9 before autoclaving. For the initial two subcultures, the medium was further supplemented with 0.06 μM IAA and 15 μM silver thiosulfate (STS) to encourage root growth and mitigate vitrification, respectively [28].

Ari et al. explored the irradiated pollen technique for homozygosity fixation in Cucumis melo, resulting in a 94% fruit set and a 96% germination rate, showcasing the E20A+ 0.1 mg/L IAA medium’s capacity to support rapid pure line development in plant breeding [59]. Kurtar et al. studied the role of embryo type in haploid plant regeneration for pumpkins, observing higher transformation rates in certain embryo types with E20A alone [60]. Further studies by Kurtar et al. on winter squash examined the effectiveness of generating in vitro haploid plants, highlighting factors such as genotype and timing that influence successful haploid development using E20A media in addition to 0.01 mg/L IAA [61]. Godbole et al. demonstrated the potential of irradiation in snapmelon, using an E20A medium supplemented with 2% sucrose and 0.06 µM IAA to culture cotyledonary embryos, resulting in effective parthenogenesis and fruit growth. This comparison underscores the continual search for more effective and labor-saving haploid embryo rescue methods [62]. In a study focusing on *Cucumis melo var. inodorus*, researchers explored the efficacy of irradiated-induced pollen in cultivating haploid embryos. They emphasized the efficiency of direct sowing in five different CP nutrient media, enriched with sucrose, agar, Vitamin B12, and 0.02 mg/L IAA, along with the innovative E20A medium. They highlighted the superiority of these techniques over traditional methods, particularly stressing the importance of light source inspection [63].

A similar study examined the impact of pollen irradiation dose and genotype on haploid embryo induction in bottle gourd. The results showed genotype and irradiation doses significantly influenced haploid embryo induction and fruit set ratio. Lower doses (50 and 75 Gy) led to higher fruit set ratios. Putative haploid embryos of varying shapes were induced, with arrowhead- and cotyledonary-shaped embryos exhibiting higher conversion rates into plants compared to point- and globular-shaped embryos. Vitrification was observed during in vitro culture. Effective irradiation doses for inducing haploid embryos ranged from 50–75 Gy, emphasizing the importance of genotype and irradiation dosage optimization for successful haploid embryo induction. This study was also pursued with E20A liquid media [64]. Kurtar et al. examined dihaploidization in generating F1 hybrid summer squash varieties, highlighting the cultivation of embryos on modified E20A medium. This investigation revealed the nuanced efficacy of the process, showcasing the medium’s adaptability [65]. Bagheri et al. investigated the effect of gamma-ray doses on inducing haploidy in Iranian melon cultivars, employing an E20A medium in addition to phytoagar with three different methods mentioned in the Table 2 for culturing embryos with specific morphologies, emphasizing the influence of genotype, embryo stage, and irradiation on haploid plant generation [66]. Overall, the evolution of media uses, particularly an E20A, alongside the refinement of irradiation techniques, illustrates a significant trajectory of innovation in haploid and DH production within cucurbits and beyond. The adaptability of the medium, specificity of irradiation doses, and introduction of novel methodologies have collectively enhanced plant breeding efforts. Doubled haploids in cucurbit species, particularly melon “Piel de Sapo”, were produced via in situ parthenogenesis using irradiated pollen. The process involved evaluating seven genotypes for agronomic traits and resistance to pathogens, optimizing parthenogenetic capacity, and assessing various embryo detection methods, resulting in doubled haploid lines [67].

### 4.3. Seed Dormancy Breaking

Seed viability is crucial for Cucurbitaceae crops such as squash, watermelon, cucumber, and melon, which play pivotal roles in global agriculture and nutrition due to their nutrient-rich composition. However, challenges exist in maintaining optimal storage conditions, and preserving viability is imperative. Old or improperly stored seeds often lose viability due to energy depletion and biochemical degradation. Fortunately, in vitro techniques, notably embryo rescue, offer promise by culturing embryos in sterile, nutrient-rich environments, thus rejuvenating seeds and enabling germination. Moon et al. conducted a study to investigate the germination success of 20-year-old squash (*Cucurbita pepo* L.) lines across different media, including potting mix, hydrogen peroxide water, and in vitro SR medium (gel and liquid). The findings revealed that hydrogen peroxide inhibited germination while the potting mix yielded minimal success. In contrast, in vitro conditions significantly enhanced germination rates (from 28.5% to 36.3%), with no notable difference between gel and liquid squash rescue (SR) mediums [24]. It contains E20/21 major salts 5 mL/L, minor salts 0.1 mL/L, sucrose 12 g/mL, IAA and IBA 0.01 µg/L, respectively. This underscores the potential of the SR medium in rejuvenating old squash seeds, which is beneficial for genetic resource conservation in breeding programs.

## 5. Other Factors Influencing Embryo Rescue

The studies by Custers et al., Ezura et al., and Nunez et al. [49,68,70] collectively underscore the importance of media composition, embryonic developmental stage, and genetic variances in optimizing in vitro development and embryogenesis across various Cucumis species and melon cultivars.

Custers et al. demonstrated that the developmental stage of *Cucumis sativus* L. and wild Cucumis species’ embryos significantly impacts in vitro development rates. Their findings on MS medium supplemented with kinetin, IAA, and sucrose highlighted that later-stage embryos achieved higher development success. Modifications in kinetin and sucrose levels notably enhanced outcomes for intermediate-stage embryos [49]. Ezura et al. further explored this theme by revealing varietal differences in somatic embryogenesis within melon cultivars PI 161375 and Vedrantais. Cultured on liquid MS medium with BA and 2,4-D, the stark contrast in somatic embryo production between the two cultivars illuminated the profound influence of genetic variances on embryogenic efficiency [70]. Nunez et al. expanded upon these concepts in their study on ‘Galia’ muskmelon, comparing the efficacy of E20A and E21 media in embryo rescue. Their research highlighted that embryos aged 17–30 days post-pollination exhibited the highest rescue success in both transgenic and wild-type lines. Additionally, the E21 medium yielded superior embryo survival rates, emphasizing the importance of tailored medium supplementation [68]. In another investigation into optimal media for *C. melo* embryo rescue, Nunez et al. demonstrated the effectiveness of a sequential media strategy, where harvested embryos were initially cultured on E21 medium for germination, followed by their development on a half-strength E21 medium [20]. This approach effectively supported both the rescue and subsequent development of seedlings, illustrating the potential of adjusting media formulations (Table 1) over different developmental stages to maximize in vitro growth and development.

Although the precise mechanisms underlying interspecific and intergeneric hybridization remain incompletely understood, these processes often give rise to post-zygotic barriers. Notably, the absence of endosperm in the embryo can lead to developmental failures and subsequent abortions [71,72]. In this context, embryo rescue (ER) procedures have emerged as invaluable tools for surmounting such barriers across a diverse spectrum of plant species.

The efficacy of ER is contingent upon a multitude of factors, encompassing both procedural and environmental variables. Key considerations include the precision of the excision procedure, the maintenance of embryo integrity throughout the process, the efficacy of sterilization protocols, and the composition of the culture medium. Moreover, environmental factors such as light intensity, spectral quality, and temperature regimes exert significant influences on ER outcomes.

Intrinsic factors also exert pronounced effects on ER efficacy, including embryo size, developmental stage, and the genetic makeup of the crop under consideration [17,73,74,75]. Notably, younger embryos may present more challenges than differentiated ones, with later-stage embryos, particularly during the autotrophic phase, exhibiting higher success rates [76]. Additionally, studies have shown that torpedo-stage embryos boast the highest success rates [16].

Ensuring aseptic conditions throughout the procedure is paramount, underscoring the necessity of maintaining sterile environments, as validated by research advocating for the potential utility of incorporating antibiotics [25]. The broad scope of applications for embryo rescue (ER) extends to enhancing seed physiology, viability, and dormancy, as well as delving into investigations surrounding ploidy and chromosome elimination. This versatility underscores ER’s pivotal role in advancing various facets of plant biology, including the development of interspecific hybrids, haploid production, and germplasm conservation.

## 6. Conclusions

Cucurbitaceae stands out as one of the most globally valued and widely adapted vegetable crops. Genetic variability plays a pivotal role in crop improvement, enabling the development of desired traits. However, achieving this variability often necessitates interspecific or intergeneric crossing, leading to the common occurrence of immature seeds due to pre- and post-zygotic barriers. These barriers encompass factors such as pollen viability, stigma receptivity, chromosomal instabilities, and endosperm development issues, all of which can significantly impact hybridization success.

Embryo rescue techniques become indispensable to salvage these immature embryos, with various factors influencing their efficacy. Critical considerations include the choice of media, hormone combinations, culture conditions, and the stage of embryos. While earlier studies predominantly utilized E20/E21 media, recent advancements have seen the adoption of MS media. Optimal results have been observed with a combination of auxin and cytokinin hormones, notably 0.1 mg/L Kinetin/BAP/coconut water and 0.01 mg/L IAA, applied to immature embryos, particularly those at the heart or globular shape stages. The timing of embryo rescue is another crucial determinant of hybridization success, with the optimal window for embryo excision typically falling between 7 to 14 days post-pollination, albeit varying with each specific cross. Advanced genomic tools have become indispensable in identifying and overcoming these barriers, offering detailed insights at the molecular level. Techniques such as real-time PCR are now employed to accurately determine the developmental stage of embryos, ensuring that rescue efforts are timed to maximize viability.

Genetic compatibility and stability of hybrids are pivotal for successful breeding endeavors. Innovations in genomic selection and gene editing technologies, such as CRISPR/Cas9, are increasingly utilized to predict and enhance the stability and viability of hybrids. These advanced techniques accelerate the development of stable, viable hybrids by precisely modifying genetic material to desired traits.

In this review, we have comprehensively addressed the various issues concerning embryo rescue techniques. These techniques hold promise for broader applications in cucurbit crop research, including studies focusing on cytoplasmic male sterility and synthetic seed production. By applying similar principles of embryo rescue, researchers can effectively address challenges and unlock the potential of these areas. This cross-applicability underscores the versatility and importance of embryo rescue techniques beyond their immediate context, offering valuable insights and solutions for broader agricultural research and crop improvement efforts.

## Figures and Tables

**Table 1:** Hybridization-based embryo rescue studies across the Cucurbitaceae family.

S No	Crop 1	Crop 2	Application/Trait	Medium Used	Used Hormones	Reference
1.	*C. ficifolia,* *C. martinezii*	*C. pepo* (Queen F1), *C. pepo* (MHTC77 F 1)	Hybridization	MS medium	MS medium supplemented with 0.1 mg/L kinetin and 0.01 mg/L indol acetic acid (IAA)	[22]
2.	*C. maxima*, *C. pepo*, *C. ficifolia*, *C. maxima*, *C. argyrosperma*	*C. pepo*, *C. moschata*, *C. maxima*	Hybridization	MS basal medium	0.1 mg/L kinetin, 0.01 mg/L indole-3-acetic acid and sucrose increased to 20 g/L	[47]
3.	*C. pepo*	*C. moschata*	Hybridization	MS medium	0.1 mg/Lkinetin, 0.01 mg/L indole-3-acetic acid	[25]
4.	*Cucumis anguria*	*C. zeyheri*	Hybridization	(1) (MS + 20 mg dm^−3^ ascorbic acid) (2) (MS + 100 mg dm^−3^ casein hydrolysate) (3) (MS + 0.3 mg dm^−3^ (GA) (4) (MS + 5% coconut water)	BAP; 0.01 mg dm^−3^ IBA; 0.01 mg dm^−3^,	[48]
5.	*C. melo*		Hybridization	E21 Medium	Putriscine, glutamine, coconut water	[20]
7.	Wild *Cucurbita martinezii* Bailey (the male parent)	Domesticated *C. pepo* L. (the female parent	Hybridization	Murashige and Skoog medium	0.01 mg/L IAA and 0.1 mg/L KIN	[18]
8.	*Cucurbita pepo* L—squash		Germplasm rescue	(1) Potting mix with osmocotte. (2) Squash rescue medium	It contains E20/21 major salts 5 mL/L, minor salts 0.1 mL/L, sucrose 12 g/mL, IAA and IBA 0.01 µg, respectively	[24]
9.	*Cucumis sativus* L. and wild species.	*C. zeyheri* 2 x Sond. and *C. metuliferus Naud*	Hybridization	MS medium.	0.1 mg/L kinetin, 0.01 mg/L (IAA), and 3.5% sucrose	[49]
10.	*C. hystrix*	*C. sativus*	Hybridization	MS medium	Hormone-free solid media with 3% sucrose P.H.6	[46]
11.	*C. pepo*	*C. moschata*	Hybridization (Hull-less seed production)	MS media	0.01 IAA mg/L and 0.1 Kinetin mg/L	[21]

**Table 2:** Haploidization-based embryo rescue studies across the Cucurbitaceae family.

S No	Crop 1	Crop 2	Application/Trait	Medium Used	Used Hormones	Reference
1.	Galia muskmelon male parental line	transgenic and wild type	Haploid production Developed male parental line	Basic E20 basic medium and E21A medium with six new supplements	0.01 mg/L Indole acetic acid, 0.01 mg/L BAP, 5% coconut water	[68]
2.	*Cucumis sativus* L.		Haploid production	E20H8 medium	E20 supplemented with 7.9 mM CaCl_2_·2H_2_O, 0.17 mM CoCl_2_·6H_2_O, 0.10 mM FeEDTA, 20 g/L sucrose, and 8 g/L Bacto agar. pH 5.9.	[28]
3.	Cucurbits		Haploid production	E20A MS medium	BAP + Kin + IBA 2 + 1 + 0.5) mg/L	[69]
4.	*C. moschata*	Duchesne ex. Poir—pumpkin	Haploid	E20A medium	No hormones used	[60]
5.	Melon genotypes Y2 and Y3		Haploid production	CP nutrient media, E20A medium	vitamin B12, 0.02 mg/L IAA	[63]
6.	Winter squash (*C. maxima* Duchesne ex Lam.)		Haploid production	E20A medium	Along with media 0.01 mg/L IAA	[61]
7.	Cucumber	(Pol10, Rubin, Stawko)	Haploid production	E20A medium	Modified MS media	[58]
8.	Snapmelon (Cucumis melo var. momordica)		Haploid production	E20A medium	Supplemented with 2% sucrose and 0.06 µM IAA	[62]
9.	*Iranian melon cultivars*		Haploid production.	E20A medium.	Embryo rescue was used with three methods: direct, liquid, and integrated.	[66]
10	*Cucumis sativus* L.		Haploid production	E20A medium		[56]
11.	Cucumber	Genotype soltar and monarch	Haploid production	E20A liquid medium	Solid medium also	[23]
12.	*C. melo*		Hapolid production	MS media and E20A medium	MS medium with 0.01% IAA	[59]
13.	Summer squash (*C. pepo*)	14 varieties used	Dihaplodization.	Modified E20A medium	Only media	[65]
14.	*C. melo* L.		Haploid and double haploid production against multiple virus resistance	E20A medium	Initially liquid media and further solid media	[57]
15.	*Lagenaria siceraria* (Malign) Stanley		Haploid embryo production	E20A medium	E20A Liquid medium	[64]
16.	Cucumber	Daminus) and two greenhouse cultivars (Rubah, RZ	Haploidization	E20A medium	No hormones used	[55]
17.	PI 161375 (*Cucumis melo* L. subsp. agrestis, chinensis group)	Vedrantais (*C. melo* L. subsp. melo, cantalupensis group	Somatic embryogenesis	Embryo-induction (EI) medium	0.1 mg/L BA and 2 mg/L 2,4-D	[70]

## Data Availability

No new data were created or analysed in this study.
